# Social adversity and epigenetic aging: a multi-cohort study on socioeconomic differences in peripheral blood DNA methylation

**DOI:** 10.1038/s41598-017-16391-5

**Published:** 2017-11-24

**Authors:** Giovanni Fiorito, Silvia Polidoro, Pierre-Antoine Dugué, Mika Kivimaki, Erica Ponzi, Giuseppe Matullo, Simonetta Guarrera, Manuela B. Assumma, Panagiotis Georgiadis, Soterios A. Kyrtopoulos, Vittorio Krogh, Domenico Palli, Salvatore Panico, Carlotta Sacerdote, Rosario Tumino, Marc Chadeau-Hyam, Silvia Stringhini, Gianluca Severi, Allison M. Hodge, Graham G. Giles, Riccardo Marioni, Richard Karlsson Linnér, Aisling M. O’Halloran, Rose A. Kenny, Richard Layte, Laura Baglietto, Oliver Robinson, Cathal McCrory, Roger L. Milne, Paolo Vineis

**Affiliations:** 1 Italian Institute for Genomic Medicine (IIGM, former HuGeF), Via Nizza 52 –, 10126 Turin, Italy; 2 Department of Medical Sciences – University of Turin, C.So, Dogliotti, 14 - 10126 Italy; 3 Cancer Epidemiology & Intelligence Division, Cancer Council of Victoria, 615 St Kilda Road, Melbourne, Victoria, 3004 Australia; 4 Centre for Epidemiology and Biostatistics, Melbourne School of Population and Global Health, The University of Melbourne, Parkville Victoria, 3010 Australia; 5 Department of Epidemiology and Public Health – University College London, 1-19 Torrington Place, London, WC1E 6BT United Kingdom; 6 Institute of Evolutionary Biology and Environmental Studies, University of Zürich, Winterthurerstrasse 190, Zürich, Switzerland; 7 Institute of Biology, Medicinal Chemistry, and Biotechnology, National Hellenic Research Foundation, Leof. Vasileos Konstantinou 48, Athens, 116 35 Greece; 8 Fondazione IRCCS – Istituto Nazionale dei Tumori, Via Venezian 1, 20133 Milan, Italy; 9 Istituto per lo Studio e la Prevenzione Oncologica (ISPO Toscana), Via Cosimo Il Vecchio, 2, 50139 Florence, Italy; 10 Department of Clinical Medicine and Surgery, University of Naples Federico II, Corso Umberto I, 40, 80138 Naples, Italy; 11 Piedmont Reference Centre for Epidemiology and Cancer Prevention (CPO Piemonte), Via Santena 7, 10126 Turin, Italy; 12 Cancer Registry and Histopathology Department, ‘Civic – M. P. Arezzo’ Hospital, ASP Ragusa, Piazza Igea, 1, 97100 Ragusa, Italy; 13 MRC-PHE Centre for Environment and Health, Imperial College London, St. Mary’s Campus Paddington, W2 1PG London, United Kingdom; 14 Institute of Social and Preventive Medicine Lausanne University Hospital (CHUV), Rue du Bugnon 46, 1011 Lausanne, Switzerland; 15 Inserm U1018, Center for Research in Epidemiology and Population Health (CESP), Gustave Roussy Institute, 114 rue Edouard Vaillant, 94805 Villejuif Cedex, France; 16 Centre for Genomic and Experimental Medicine – University of Edinburgh, Crewe Road, EH4 2XU Edinburgh, United Kingdom; 17 Department of Complex Trait Genetics, Center for Neurogenomics and Cognitive Research (CNCR), Neuroscience Campus Amsterdam (NCA), VU University Amsterdam, De Boelelaan, 1085-1087 1081 HV Amsterdam, The Netherlands; 18 Trinity College Dublin, Dublin, Ireland UK; 19 Department of Clinical and Experimental Medicine, University of Pisa, Lungarno Antonio Pacinotti, 43, 56126 Pisa, Italy

## Abstract

Low socioeconomic status (SES) is associated with earlier onset of age-related chronic conditions and reduced life-expectancy, but the underlying biomolecular mechanisms remain unclear. Evidence of DNA-methylation differences by SES suggests a possible association of SES with epigenetic age acceleration (AA). We investigated the association of SES with AA in more than 5,000 individuals belonging to three independent prospective cohorts from Italy, Australia, and Ireland. Low SES was associated with greater AA (β = 0.99 years; 95% CI 0.39,1.59; p = 0.002; comparing extreme categories). The results were consistent across different SES indicators. The associations were only partially modulated by the unhealthy lifestyle habits of individuals with lower SES. Individuals who experienced life-course SES improvement had intermediate AA compared to extreme SES categories, suggesting reversibility of the effect and supporting the relative importance of the early childhood social environment. Socioeconomic adversity is associated with accelerated epigenetic aging, implicating biomolecular mechanisms that may link SES to age-related diseases and longevity.

## Introduction

Lower socioeconomic status (SES) is associated with lower life expectancy and earlier onset of age-related chronic conditions, based on robust worldwide evidence from studies using a range of SES and health indicators^1–4^. A recent study investigating the association of low SES with health in more than 1.7 million individuals, confirmed low SES as an independent predictor of premature mortality, with an associated effect size comparable to, and independent of that of the main non communicable disease (NCD) risk factors, including smoking, physical inactivity, higher alcohol intake, obesity, and hypertension^5^. Although SES is an overarching health determinant, with NCD risk factors unevenly distributed between SES strata, SES-health associations are only partly explained by the unhealthy lifestyle habits of individuals with lower SES^5,6^.

Despite extensive research efforts, the biological mechanisms that mediate the impact of SES on age-related conditions are still not fully understood. Systemic inflammation and immunological impairment in response to psychosocial stressors have been proposed as mechanisms through which SES is biologically embedded. Pivotal studies in macaques detected altered levels of expression and methylation in inflammatory and immune function-related genes depending on dominance rank (a proxy for SES), which were reversible following changes in dominance rank^7,8^. In humans, low SES has been associated with higher inflammatory status, the latter measured using epigenetic^9–12^, transcript^13^, and protein^14,15^ biomarkers. Interestingly, SES in early life appears particularly important in predicting higher inflammation status later in life^14^. Recent studies have employed multi-biomarker indicators of physiological function such as allostatic load, which incorporates markers of stress response, inflammation, and cardio-metabolic function, to link social adversity to health status^4,16,17^.

Accelerated biological aging, which may be measured through a multiple biomarker approach, is likely to be the result of several different physiological and pathological changes during the life-course, and therefore may represent an overarching mechanism linking SES and health. Recently, Horvath developed a multi-tissue predictor, that allows the age of most tissues and cell types to be estimated based on DNA methylation (DNAm) at 353 CpG sites^18^, while Hannum *et al*.^19^ developed a blood-specific DNAm age predictor based on levels of 71 CpG sites. These predictors, known as ‘epigenetic clocks’, allow estimation of whether an individual is experiencing accelerated or decelerated aging by defining age acceleration (AA) as the difference between DNAm age and chronological age. Recently, the ‘intrinsic’ AA defined as the residual from the regression of AA on chronological age and white blood cell (WBC) percentages, was proposed as a more reliable estimate of biological aging as it captures cell-intrinsic properties of the aging process that exhibit some conservation across various cell types and organs^20^.

The present study examines the association of SES with intrinsic AA in 5,111 adults from three large prospective cohorts: the Italian component of the European Prospective Investigation into Cancer and Nutrition (EPIC, Italy), the Melbourne Collaborative Cohort Study (MCCS, Australia), and The Irish LongituDinal study on Aging (TILDA, Ireland). We used a standardized measure of educational attainment (that is directly comparable across countries) as a proxy for SES. Further, we used a measure that incorporates SES in early life and adulthood (based on occupational position) to explore the association between SES changes across the life-course and AA. Finally, we investigated the role of NCD risk factors in modulating the SES-AA association.

## Results

### Study Populations

After pre-processing, quality controls, and sample filtering, 5,111 subjects were included in the analyses. There were several differences by study in the distribution of demographic variables and NCD risk factors (Table 1). Table 2 shows demographic and covariate information by SES categorized as ‘low’, ‘medium’ and ‘high’ (Methods). In the linear regression adjusted for study area, obesity (lower BMI in higher SES group), diet (healthier diet in higher SES group), and physical activity (lower percentage of inactive individuals in higher SES group), were associated with SES, whereas no significant differences were observed for smoking habits and alcohol consumption (Table 2).

**Table 1:** Participant characteristics: Descriptive statistics of the study participants by cohort. Mean and standard deviation are reported for continuous variables, absolute numbers and percentages of individuals in each group are reported for categorical variables.

	EPIC Italy	MCCS	TILDA	p*
N	1803	2818	490	—
Sex (males)	689 (38%)	1723 (61%)	244 (50%)	<0.0001
Age	53.3 (7.2)	59.0 (7.6)	62.1 (8.1)	<0.0001
BMI	26.2 (4.1)	27.1 (4.0)	28.6 (4.6)	<0.0001
Mediterranean diet score	4.0 (1.8)	4.7 (1.6)	—	<0.0001
Smoking				<0.0001
Current	501 (28%)	388 (14%)	86 (18%)	
Former	503 (28%)	1077 (38%)	211 (43%)	
Never	777 (44%)	1532 (48%)	193 (40%)	
Physical activity				<0.0001
Inactive	494 (28%)	617 (22%)	—	
Mod. inactive	614 (34%)	564 (20%)	142 (29%)	
Mod. active	387 (22%)	1060 (38%)	175 (36%)	
Active	286 (16%)	576 (20%)	167 (35%)	
Alcohol^+^	302 (17%)	816 (29%)	80 (18%)	<0.0001

^*^Chi-squared test for categorical variables, One-way ANOVA for continuous variables.

^+^Absolute numbers and percentages of habitual drinkers.

**Table 2:** Participant characteristics: Descriptive statistics of the study participants by SES. Mean and standard deviation are reported for continuous variables, absolute numbers and percentages of individuals in each group are reported for categorical variables.

	High SES	Medium SES	Low SES	p*
N	1744	1749	1594	—
Sex (males)	908 (52%)	971 (55%)	763 (48%)	<0.0001
Age	57.5 (7.6)	56.3 (8.9)	58.2 (7.6)	<0.0001
BMI	26.4 (4.2)	27.3 (4.2)	27.1 (4.1)	0.03
Mediterranean diet score	4.6 (1.8)	4.5 (1.7)	4.3 (1.7)	<0.0001
Smoking				
Current	289 (17%)	401 (23%)	285 (18%)	0.27
Former	623 (36%)	632 (36%)	535 (34%)	0.25
Never	832 (48%)	716 (41%)	774 (49%)	0.38
Physical activity				
Inactive	316 (18%)	431 (25%)	364 (23%)	0.0007
Mod. inactive	484 (28%)	462 (26%)	374 (23%)	0.001
Mod. active	533 (31%)	592 (34%)	496 (31%)	0.40
Active	409 (23%)	261 (15%)	359 (23%)	0.68
Alcohol^+^	436 (26%)	399 (23%)	361 (23%)	0.62
Hannum AA^++^	−0.47 (6.0)	−0.13 (6.1)	0.35 (5.7)	<0.0001
Horvath AA^++^	−0.50 (6.7)	−0.17 (6.7)	0.43 (6.2)	<0.0001

^*^Test for linear trend adjusted by study area.

^+^Absolute number and percentages of habitual drinkers.

^++^Intrinsic AA: residual from the regression of AA on chronological age and white blood cell (WBC) composition.

### Age Acceleration (AA) measures

We estimated the epigenetic age of each blood sample using both the approach by Horvath based on 353 CpGs^18^, and the approach by Hannum *et al*. based on 71 CpGs^19^. Out of the 418 age-related CpGs (347 specific for the Horvath measure, 65 specific for the Hannum measure, and 6 CpGs that are in common between the two indicators), we detected 414 CpGs (99%) in EPIC, 416 (99.5%) in MCCS, and 396 (95%) in TILDA. The CpGs identified in EPIC and MCCS but not in TILDA are those that are not present in the new *Illumina 850k methylation BeadChip*, that has been used for the Irish cohort only (see Supplementary Methods). All the probes had less than 5% missing in the study sample. For the SES-AA associations, we used the epigenetic age measures computed after imputation of missing data, after verifying the concordance with those obtained without the imputation procedure (Pearson correlation coefficients > 0.99; p < 2 × 10^−16^).

Both DNAm age estimates were highly correlated with chronological age (Pearson correlation coefficients ranging from 0.73 to 0.80). Also, the Horvath and Hannum estimates were highly correlated with each other (Pearson correlation coefficients ranging from 0.80 to 0.92; Figure S1).

Hannum DNAm age was a slightly better predictor of chronological age than Horvath DNAm age. The Pearson correlation coefficients ranged from 0.73 to 0.77 for the Horvath measure, and from 0.74 to 0.80 for the Hannum measure. The average absolute difference (± standard deviation) between DNA methylation predicted age and chronological age was 4.03 ± 3.56 for Horvath measure, and 3.72 ± 3.25 for Hannum measure. Given the above, and since Hannum AA is more specific for DNA methylation in blood cells, we present associations of SES with the Hannum intrinsic AA (referred to hereafter as merely ‘AA’) in the main text. Associations with Horvath intrinsic AA are presented in the supplementary material.

Table 3 shows the results for the association of NCD risk factors with intrinsic AA measures. All the NCD risk factors with the exception of physical activity were significantly associated with epigenetic aging, AA being higher in men, obese people, smokers, habitual alcohol drinkers, and individuals with unhealthy diets.

**Table 3:** NCD risk factors-AA associations: Linear regression models with age acceleration (AA) as the outcome and NCD risk factors as the predictors adjusted by study area. For categorical variables (sex, smoking, physical activity, and alcohol), the effect sizes (β) are interpretable as years of increase/decrease epigenetic age compared with the reference group. For continuous variables (BMI and Mediterranean diet score), the effect sizes (β) are interpretable as years of increase/decrease epigenetic age for each unit increase of the predictor.

	Hannum intrinsic AA		Horvath intrinsic AA	
	β (95% CI)	p	β (95% CI)	p
Sex^+^	−1.95 (−2.28, −1.62)	<0.0001	−1.76 (−2.12, −1.39)	<0.0001
BMI	0.09 (0.05, 0.13)	<0.0001	0.09 (0.05, 0.14)	<0.0001
Mediterranean diet score	−0.11 (−0.21, −0.01)	0.03	−0.07 (−0.18, 0.04)	0.24
Smoking^++^				
Former	−0.63 (−1.10, −0.16)	0.01	−0.27 (−0.79, 0.25)	0.31
Never	−1.48 (−1.93, −1.03)	<0.0001	−1.32 (−1.82, −0.83)	<0.0001
Physical activity^+++^				
Mod. inactive	0.03 (−0.45, 0.51)	0.90	0.05 (−0.48, 0.57)	0.86
Mod. active	0.14 (−0.32, 0.60)	0.55	−0.12 (−0.63, 0.39)	0.64
Active	0.11 (−0.40, 0.63)	0.67	0.17 (−0.39, 0.74)	0.55
Alcohol^++++^	0.76 (0.37, 1.15)	0.0001	0.73 (0.31, 1.16)	0.001

^+^Reference: Men.

^++^Reference: Current smokers.

^+++^Reference: Inactive.

^++++^Reference: No/moderate drinkers.

Chronological age is not associated with the intrinsic AA by definition.

### SES – AA association

**Figure 1:**
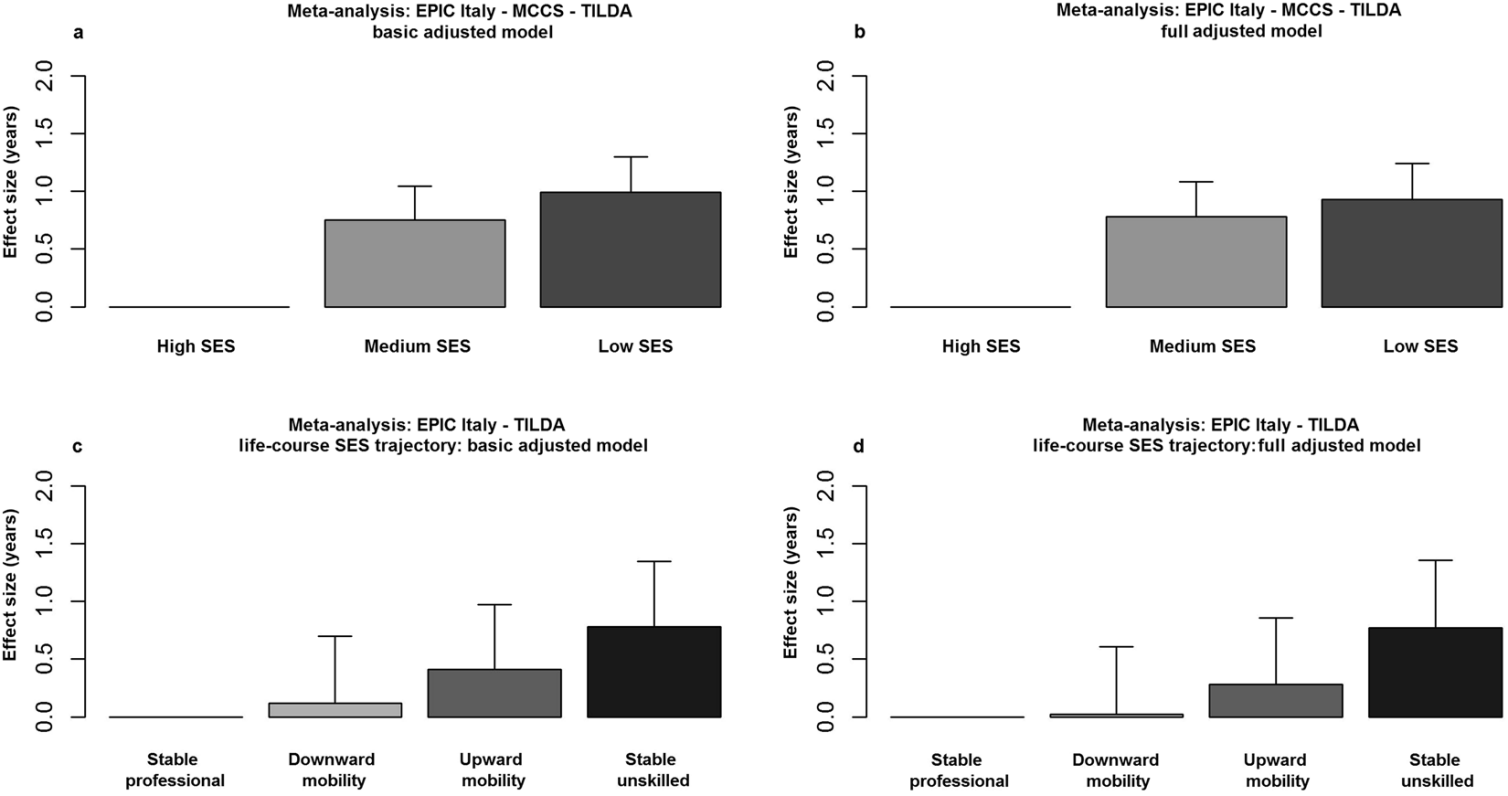
SES–AA association: Bar-plots indicating the estimated effect sizes (in years) and standard errors of the association of SES with Hannum AA (**a**: model 1 with basic adjustments, **b**: model 2 adjusted for NCD risk factors), and life-course SES trajectory with Hannum AA (**c**: model 1 with basic adjustments, **d**: model 2 adjusted for NCD risk factors).

In the meta-analysis of the three study results, SES was associated with AA (Table 4, Fig. 1a) in the basic adjusted model. The effect sizes (interpretable as years of increase in epigenetic age) were β = 0.75 (95% confidence interval (CI): 0.17, 1.34; p = 0.01) and β = 0.99 (95% CI: 0.39, 1.59; p = 0.001) comparing participants of medium SES and low SES with those of high SES, respectively. The estimated linear trend of increased AA per decrease in SES was β = 0.42 (95% CI: 0.15, 0.68; p = 0.002). Similar effect estimates were observed in the single cohorts, with the association being significant in EPIC Italy and MCCS (Table 4). Comparable results were observed using Horvath intrinsic AA as the outcome (Table S1), as well as using alternative SES indicators like the index of relative socio-economic disadvantage (IRSD): β = 0.91 (95% CI: 0.21, 1.62; p = 0.01, Table S6), and household income: β = 0.66 (95% CI: −1.03, 2.35; p = 0.45, Table S7). Further, we show that different SES indicators are strongly associated among themselves (Table S8).

**Table 4:** SES–AA association: By study area and showing overall meta-analysis of the three study results. Linear regression models with age acceleration (Hannum intrinsic AA) as the outcome and SES as the predictor. Regression models included age, gender, center of recruitment (EPIC Italy and TILDA), case-control status (EPIC Italy only), and sample type (MCCS only).

SES	N	β (95% CI)	p	I^2^
**EPIC Italy**				
High	624	0.00 (reference)	—	
Medium	643	0.82 (0.07, 1.57)	0.03	
Low	514	1.03 (0.29, 1.77)	0.01	
Linear trend	1781	0.41 (0.09, 0.74)	0.01	
**MCCS**				
High	952	0.00 (reference)	—	
Medium	948	0.46 (−0.31, 1.07)	0.13	
Low	917	0.84 (0.17, 1.51)	0.01	
Linear trend	2817	0.40 (0.09, 0.71)	0.01	
**TILDA**				
High	168	0.00 (reference)	—	
Medium	158	1.06 (−0.63, 2.75)	0.22	
Low	163	1.03 (−0.72, 2.79)	0.25	
Linear trend	489	0.52 (−0.36, 1.39)	0.25	
**Meta-analysis**				
High	1744	0.00 (reference)	—	—
Medium	1749	0.75 (0.17, 1.34)	0.01	0
Low	1591	0.99 (0.39, 1.59)	0.001	0
Linear trend	5087	0.42 (0.15, 0.68)	0.002	0

*I^2^ statistic indicates the percentage of variance that is attributable to study heterogeneity.

### Basic adjusted model comparison with NCD risk factors adjusted model

We evaluated the change in the magnitude of the SES-AA association due to the inclusion of NCD risk factors in the model, by comparing the estimated effects (βs) of the basic regression model (adjusted for age and sex) with those of the models including different NCD risk factors. NCD risk factors considered were smoking status, BMI, alcohol intake, Mediterranean Diet Score, and physical activity. Additional covariates were first added to the basic adjusted model one-by-one and finally included all together in the ‘fully adjusted’ model (Table 5).

**Table 5:** Reduction of SES–AA (Hannum intrinsic AA) association by non-communicable disease (NCD) risk factor: Effect size reduction percentage due to the inclusion of NCD risk factors in the model (right side of the table) was computed as 1-β_m_/β_1_; where β_1_ is the effect size of model 1 (basic adjustments, on the top of the table) and β_m_ is the effect size of model 1 + risk factor (model 2, left side of the table). Negative attenuations indicate increased effect size in model 2 (positive confounding). For attenuation percentages, confidence intervals (CIs) and p-values were estimated using a block jackknife procedure based on 1,000 resampling.

**SES**	**β**_**1**_ **(95% CI)**	**p**	**% Attenuation (95% CI)**	**p**
**Meta-analysis (basic adjusted model)**				
Medium	0.75 (0.17, 1.34)	0.012	—	—
Low	0.99 (0.39, 1.59)	0.001	—	—
Linear trend	0.46 (0.19, 0.73)	0.001	—	—
**Meta-analysis (basic adjusted model + BMI)**				
Medium	0.76 (0.17, 1.35)	0.011	−1 (−13, 11)	0.86
Low	1.01 (0.41, 1.61)	0.001	−2 (−18, 13)	0.79
Linear trend	0.45 (0.18, 0.73)	0.001	2 (−12, 17)	0.77
**Meta-analysis (basic adjusted model + alcohol)**				
Medium	0.83 (0.24, 1.43)	0.006	−11 (−23, 2)	0.10
Low	1.03 (0.43, 1.64)	0.001	−4 (−22, 13)	0.61
Linear trend	0.47 (0.20, 0.74)	0.001	−2 (−15, 11)	0.77
**Meta-analysis (basic adjusted model + physical activity)**				
Medium	0.76 (0.17, 1.35)	0.012	0 (−15, 15)	0.97
Low	0.98 (0.37, 1.58)	0.002	1 (−11, 0.13)	0.85
Linear trend	0.46 (0.19, 0.73)	0.001	1 (−12, 0.14)	0.90
Meta-analysis (basic adjusted model + Mediterranean diet score)*				
Medium	0.77 (0.15, 1.40)	0.016	−2 (−18, 13)	0.78
Low	1.02 (0.38, 1.66)	0.002	−3 (−20, 14)	0.72
Linear trend	0.46 (0.18, 0.75)	0.002	0 (−13, 14)	0.95
**Meta-analysis (basic adjusted model + smoking)**				
Medium	0.74 (0.15, 1.33)	0.013	2 (−11, 0.14)	0.79
Low	0.94 (0.34, 1.54)	0.002	5 (−7, 0.17)	0.44
Linear trend	0.44 (0.17, 0.71)	0.002	6 (−11, 0.22)	0.50
**Meta-analysis (fully adjusted model)**				
Medium	0.78 (0.19–1.37)	0.01	−4 (−18, 11)	0.63
Low	0.93 (0.32–1.54)	0.003	6 (−7, 18)	0.37
Linear trend	0.41 (0.13–0.68)	0.004	12 (−2, 26)	0.09

^*^Diet not measured in the TILDA study.

None of the NCD risk factors were associated with a statistically significant reduction in the effect size for the SES-AA association. In the fully adjusted model, the decrease in the effect size was close to being significant. The effect size attenuation for the linear trend was 12% (95% CI: −0.02, 0.26; p = 0.09), suggesting that only part of the SES-AA association could be explained by these risk factors (Table 5, Fig. 1b). The most substantial contribution to the reduction of the effect size for the SES-AA association was observed when including smoking in the regression model, which attenuated the estimate for linear trend by 6% (95% CI: −11, 22). Effect size reductions due to the inclusion of other NCD risk factors were small and non-significant.

### Life-course SES trajectory – AA association

The life-course SES trajectory is based on father’s occupational position (a proxy for early life SES), and highest occupational position (a proxy for adulthood SES). It is defined as a categorical variable with four levels, corresponding to four possible SES trajectories: high SES in childhood - high SES in adulthood (stable professional, the reference group), high SES in childhood - low SES in adulthood (any downward mobility), low SES in childhood - high SES in adulthood (any upward mobility), and low SES in childhood - low SES in adulthood (stable unskilled). The measure was not available for the MCCS cohort.

In the meta-analysis of EPIC Italy and TILDA results (Table 6, Fig. 1c), we observed a positive trend of increase in AA with decreasing SES (β = 0.78, 95% CI: −0.33, 1.89; p = 0.17, comparing the extreme categories: stable unskilled and stable professional), with some attenuation after further adjustment for NCD risk factors (Table 6, Fig. 1d, β = 0.77; 95% CI: −0.37, 1.92; p = 0.19), although these associations did not reach statistical significance. Interestingly, individuals who experienced changes in SES over their lifetime (any upward or downward social mobility) had an AA that was intermediate between the stable professional and the stable unskilled groups, and AA was greater for individuals with low SES in childhood than for those with low SES in adulthood (Table 6, Fig. 1c,d).

**Table 6:** Life-course SES trajectory–AA association: Meta-analysis of EPIC Italy and TILDA results. Linear regression models with age acceleration (Hannum intrinsic AA) as the outcome and life-course SES trajectory as the predictor. Model 1 included age, gender, center of recruitment, and case-control status (EPIC Italy only); model 2 was as model 1 plus smoking status, BMI, alcohol intake, Mediterranean diet score (EPIC Italy only) and physical activity.

Life-course SES	N	β (95% CI)	p	β (95% CI)	p		
		Model 1 (basic adjusted)		Full model (adjusted for NCD risk factors)			
**EPIC Italy**							
Stable professional	48	0.00 (reference)	—	0.00 (reference)	—		
Downward mobility	317	0.34 (−1.09, 1.77)	0.64	0.40 (−1.02, 0.64)	0.58		
Upward mobility	653	0.42 (−0.96, 1.81)	0.55	0.52 (−0.86, 0.55)	0.46		
Stable unskilled	660	0.57 (−0.81, 1.95)	0.42	0.56 (−0.82, 0.42)	0.43		
Linear trend	1678	0.14 (−0.14, 0.42)	0.32	0.11 (−0.17, 0.32)	0.46		
**TILDA**							
Stable professional	123	0.00 (reference)	—	0.00 (reference)	—		
Downward mobility	121	−0.25 (−2.10, 1.61)	0.8	−0.70 (−2.66, 0.80)	0.49		
Upward mobility	125	0.39 (−1.45, 2.24)	0.68	−0.20 (−2.17, 0.68)	0.84		
Stable unskilled	121	1.17 (−0.69, 3.03)	0.22	1.24 (−0.81, 0.22)	0.24		
Linear trend	490	0.42 (−0.17, 1.00)	0.16	0.39 (−0.25, 0.16)	0.23		
**Life-course SES**	**N**	**β (95% CI)**	**p**	**I** ^**2**^	**β (95% CI)**	**p**	**I** ^2*^
**Meta-analysis**							
Stable professional	171	0.00 (reference)	—	—	0.00 (reference)	—	—
Downward mobility	438	0.12 (−1.01, 1.25)	0.84	0	0.02 (−1.13, 1.17)	0.97	0
Upward mobility	778	0.41 (−0.69, 1.51)	0.47	0	0.28 (−0.85, 1.41)	0.62	0
Stable unskilled	781	0.78 (−0.33, 1.89)	0.17	0	0.77 (−0.37, 1.92)	0.19	0
Linear trend	2168	0.19 (−0.06, 0.44)	0.14	0	0.16 (−0.10, 0.41)	0.24	0

*I^2^ statistic indicates the percentage of variance that is attributable to study heterogeneity.

The estimated effect size was greater and significant in men compared with women, with the association being significant in men when comparing the extreme categories (Table S3), though there was no evidence of SES-gender interaction (Tables S4 and S5).

### Sensitivity analysis

**Figure 2:**
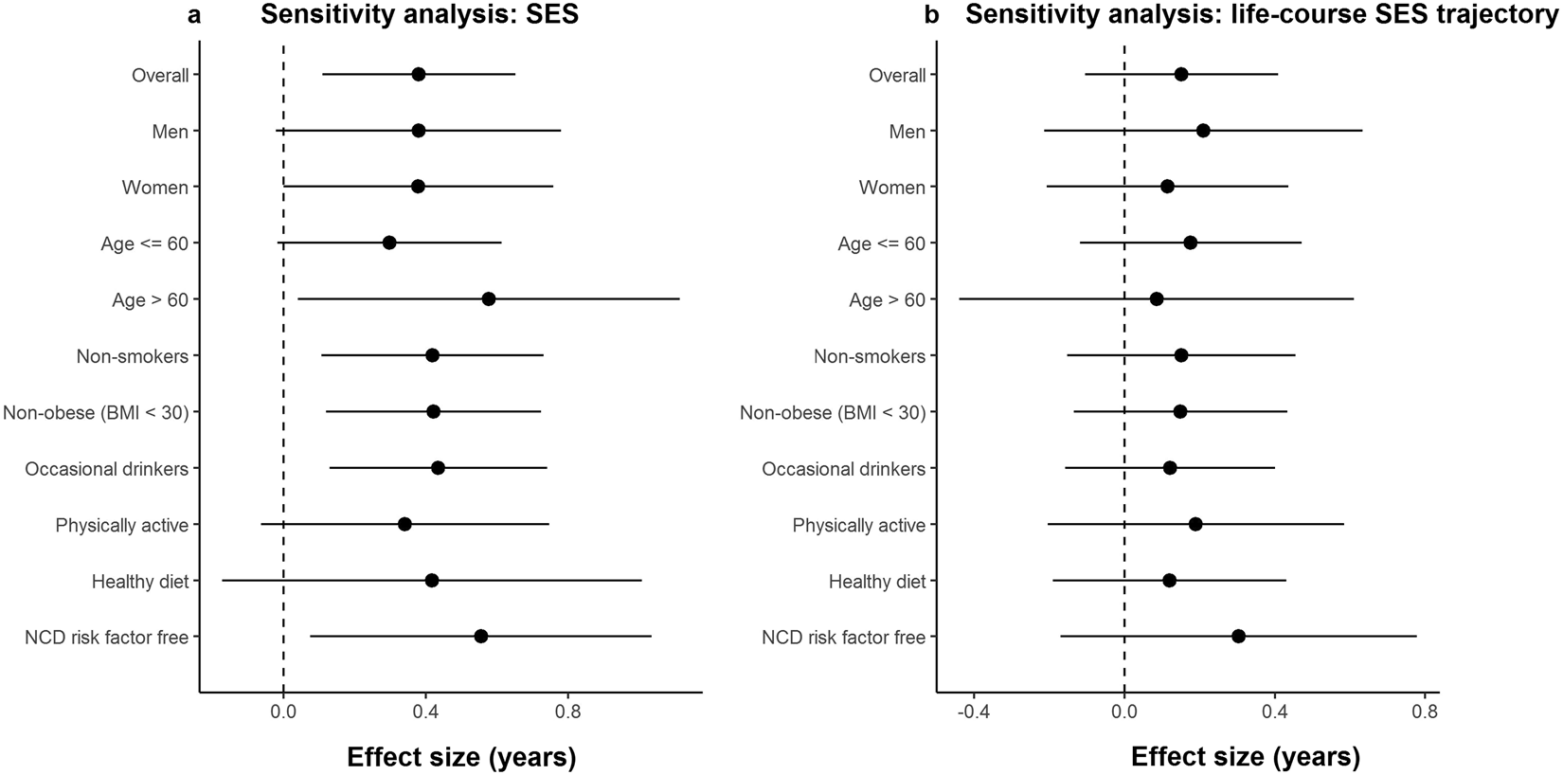
Sensitivity analysis: Forest-plots indicating the estimated effect sizes (in years; black dots) and 95% confidence intervals (horizontal lines) for the association of SES (**a**: three studies meta-analysis) and life-course SES trajectory (**b**: EPIC Italy and TILDA meta-analysis) with Hannum AA, estimated each time in different subsets of the overall sample.

Figure 2 shows repetitions of meta-analyses for SES and life-course SES trajectory stratified by sex and age (younger than, and older than 60 years old). The estimated magnitude of the association was overall consistent across genders and age groups (Fig. 2), with no evidence of SES-gender and SES-age interaction (Table S5).

Further, SES-AA associations were assessed in subsets of the overall sample, excluding each time individuals exposed to NCD risk factors: current smokers, habitual drinkers, physically inactive, obese (BMI > 30), and individuals with unhealthy diet (Mediterranean diet score <= 4) (Fig. 2). The estimated effects were comparable to those estimated on the whole sample but with lower statistical significance due to the reduced statistical power. Finally, we examined the SES-AA associations after exclusion of individuals exposed to at least one NCD risk factor and of incident cancer/cardiovascular disease cases. This procedure led to the analysis of 375 Italians, 1,106 Australians and 68 Irish NCD risk factor free individuals (non-obese, non-smokers, physically active, non-drinkers and with a healthy diet). Notably, the association of SES with AA was still significant in this NCD risk factor free subsample (test for linear trend β = 0.62; 95% CI: 0.06, 1.18; p = 0.03; N = 1,549; Fig. 2a).

#### Other SES indexes – AA association

In MCCS and TILDA cohorts we tested the association of AA with alternative SES indicators. In MCCS, AA was also significantly associated with the index of relative socioeconomic disadvantage (IRSD, Table S6) that is a general index summarizing the socioeconomic conditions of individuals within a given area defined by the Australian Bureau of Statistics^21^. In TILDA, increasing AA with decreasing income was observed, with estimates comparable to those described for the other SES variables. However, the association was not statistically significant due to the reduced statistical power (Table S7).

## Discussion

We explored the association between multiple SES indicators and epigenetic aging in three large cohort studies involving more than 5000 individuals. Lower SES was associated with accelerated epigenetic aging compared to higher SES, with in-between effects for intermediate SES.

In the three cohorts, adult SES was assessed using the highest level of educational attainment, a good proxy for SES that is usually completed before the onset of many chronic diseases, therefore reducing the risk of reverse causation^22^. Further, we have analyzed alternative SES indicators based on occupational position, household income, and a deprivation index. We investigated the association of these different SES indices with the ‘intrinsic’ AA defined as the residuals from the regression of AA on chronological age and WBC percentages^20^. According to Horvath and colleagues, this measure is a more reliable indicator of accelerated aging than ‘crude’ AA because it captures cell-intrinsic properties of the aging process that exhibit some preservation across various cell types and organs^20^. From a statistical point of view, the described procedure allows us to avoid bias due to the correlation of AA with chronological age and the estimated percentages of WBC.

The main finding of this study is the observed graded relationship between SES (variously defined) and AA. The biological (epigenetic) age of low SES individuals was estimated to be on average one year higher than for high SES subjects. We obtained consistent results using two alternative SES indicators: the IRSD and the household income (Tables S6 and S7), due to the high association between different SES indicators (Table S8). According to a recent meta-analysis investigating the association of epigenetic age with mortality in more than 13,000 individuals^20^, our estimate corresponds to an increased risk of death in the monitored time period (around 11 years of follow up on average) with a hazard ratio (HR) ranging between 1.01 and 1.04, depending on the AA measure.

The increased AA associated with low SES was lower than that estimated for smoking (1.5 years increase in AA for current smokers compared with never smokers), and comparable to that estimated for obesity (one year increase in AA per 10 unit increase in BMI), unhealthy diet (one year increase in AA comparing Mediterranean diet score extreme categories), and alcohol consumption (0.75 years increase in AA for habitual alcohol consumers compared to no/moderate consumers). These results are consistent with our recent meta-analysis of 48 independent cohorts involving more than 1.7 million subjects^5^, that estimated a two year reduction in life expectancy for low SES compared to high SES individuals, with an effect comparable to those of other risk factors.

### SES trajectory and reversibility of the effect

Our results also suggest that the relationship between SES and AA may be responsive to early life social influences. Recent research shows that effects of *in-utero* and early life exposures (including those associated with SES) may be stored in cells through epigenetic modifications that can be sustained for decades^23–26^. It can be speculated that long-term effects of early life exposures may be due to their impact on somatic stem cell populations, which persist as a form of cellular memory, including changes in DNAm patterns. One of the aims of this study was to investigate whether the association of SES with AA is reversible. It was possible to address this question in the Italian and Irish cohorts using the cross-classification of childhood and adulthood social class (early life SES was not available for the MCCS cohort). Our results suggest that AA for individuals who experienced downward mobility (high childhood SES, low adulthood SES) is more comparable to that of the stable professional group, and lower than that for the upwardly mobile (low childhood SES, high adulthood SES), supporting previous observations of the relative importance of the early childhood social environment^27–29^. Moreover, AA was higher for individuals whose childhood SES was low and remained low in adulthood compared with those experiencing upward mobility (low SES in childhood, high SES in adulthood). This pattern is consistent with some degree of reversibility of the unfavorable effect of childhood social adversity.

### The role of NCD risk factors in modulating SES-AA association

Due to the relationship of NCD risk factors with both SES and AA (Tables 2 and 3), we evaluated regression models adjusted for potential confounders, and then sequentially adjusted for mediators, to assess the change in the estimated effect of SES on AA. The concepts of ‘confounder’ and ‘mediator’ are often misinterpreted, particularly when referring to SES, which is an overarching determinant of health. Mediators and confounders are similar except for the direction of the relationship between them and the main exposure, in this case, SES^30^. In our analyses, chronological age and sex were potential confounders because they influence SES (not *vice versa*) and affect the outcome (AA), but they do not belong to the causal pathway between SES and health. Instead, lifestyle-related behaviors (smoking habits, BMI, alcohol intake, physical activity, and diet) have to be considered as mediating factors as they are influenced by SES and are simultaneously major risk or protective factors for health-related outcomes. They contribute to the SES-AA association by being located on the presumed causal pathway between SES and health^5^.

In our analyses, we did not observe a significant reduction of the association magnitude due to the inclusion of mediators in the regression model. The associations were robust to adjustment for mediators, although with slightly reduced effect size. The most significant contribution to the partial effect attenuation was observed when including smoking in the statistical model. This pattern of findings suggests that only part of the observed association between SES and AA could be explained by unhealthy lifestyle habits of individuals with lower SES, with smoking being one of the most significant mediators. The residual association is likely attributable to an altered inflammatory status, that is known to be associated at the same time with unhealthy lifestyle (e.g. smoking, poor physical activity)^31,32^, lower SES^14^, and accelerated aging^33^. Further, health inequalities across different SES groups have been explained with disparities in the allostatic load (AL) index^17,34^. AL is a commonly used metric of health based on the hypothesis that multiple exposures to stressors lead to a progressive dysregulation of different physiological systems^34^. Disparities in AL have been described as a consequence of childhood social adversities^16,35^, further supporting our findings on the relative importance of the early life social environment.

We conducted extensive sensitivity analyses confirming the SES-AA association in different subsets of the overall sample and using different SES indicators. Further, to rule out potential ‘collider bias’^36^, we verified the association in the subgroup of NCD risk factor free individuals (non-obese, non-smokers, non-drinkers, physically active, with a healthy diet, and non-incident cancer/cardiovascular cases; N = 1,549).

### Strengths and limitations

Our study has some limitations. The Italian study sample is enriched for incident cases of breast, colon, and lung cancers, lymphoma, and myocardial infarction (though blood samples were collected several years before the disease diagnosis), and the Australian study sample included 11% of controls that were matched to lung cancer cases for smoking status, leading to a slight over-representation of smokers. Conversely, the sample from the Irish TILDA cohort is representative of the national population aged 50 years and over, but the subgroup selected for these analyses was explicitly chosen to examine social mobility associations with AA with the four life-course SES trajectories being equally represented. While most NCD risk factors were measured with the same degree of accuracy as SES, diet is more difficult to measure. No measure of diet comparable to the Mediterranean diet score was available in the TILDA cohort. Therefore we may not have been able to thoroughly assess the contribution of diet as a mediator in the SES-AA association. Also, further investigation is needed to evaluate the role of inflammation in modulating the SES-AA association properly.

Although the association of SES with epigenetic aging was already described in two independent Afro-American cohorts^37,38^, these studies were based on limited samples of women (N = 100) and teenagers (N = 292). Further, extrinsic epigenetic AA, but not intrinsic epigenetic AA was associated with educational attainment and income in a cross-sectional study involving 4,173 postmenopausal women^33^. Epidemiology is based on an accumulation of evidence, and the extent to which SES is linked to AA has not been fully investigated in other populations until now. Also, we provide further evidence of the relative importance of the early life social environment, and suggest some degree of reversibility of epigenetic changes, as observed more prominently for smoking-associated methylation^25^, which has important policy implications.

### Policy implications

The implications of long-lasting impacts of life experiences, and particularly SES, on the modulation of epigenetic variations are vast, particularly concerning their public policy significance. While most policies targeted at poverty are focused on adults, such as the unemployed or workers with low incomes (for example, the Earned Income Tax Credit program in the USA), some of these policies also cover infancy. Examples of the latter are Conditional Cash Transfer programs that incentivize schooling and health programs for children^39^.

Our findings in the Italian and Irish cohorts suggest thatearlier interventions are likely to pay greater dividends than interventions later in life. A related question is whether and to what extent epigenetic changes are reversible, and if they are, which targeted interventions could be most beneficial. Both our previous work^12,15^ and the current study suggest that the relationship between SES and methylation is more pronounced in individuals whose SES starts low and remains low later in life, compared to those whose status improves during the life-course, while intermediate changes were found in subjects whose SES declined from high to low. This pattern of findings is consistent with some degree of reversibility.

## Conclusions

To our knowledge, this is the largest study investigating the relationship between SES and DNAm age (the ‘epigenetic clock’) in peripheral blood in adults. Our results confirm previous observations that SES is a determinant of health that goes beyond the major risk factors for diseases and may involve independent biological mechanisms^37,40^. Also, our findings support the hypothesis of a life-course accumulation of exposures and suggest some degree of reversibility of the effect, which has significant policy implications. More generally, our approach based on epigenetic measurements may contribute to the identification of SES-specific mechanisms that influence aging and health.

## Methods

Details on subject recruitment and relevant demographic and lifestyle variables acquisition are presented in supplementary text.

### Socioeconomic status assessment

In all cohorts, the highest level of educational attainment was used as a proxy for SES. To avoid bias due to the different proportions of educational qualifications by gender, birth cohort and study centers, a standardized version education was computed as follows: 1) the highest educational attainment was categorized as primary school or none, vocational or another secondary school, and university or vocational postsecondary school; 2) individuals were grouped for gender, center of recruitment, ethnicity (in MCCS only as Mediterranean or Anglo-Saxon) and 10-year age groups; 3) for each group the proportion of individuals in each educational level was computed; 4) a score was computed by taking into account the distribution of educational level in each group. For example, if within a given group 60% of participants were in the higher educational level, 30% were in the middle educational level and 10% were in the lower educational level, in that group each individual in the higher educational level would be assigned a score of 0.30 (0.60/2), each participant in the intermediate stratum would be assigned a score of 0.75 (0.60 of the first level, plus 0.30/2), and finally, the remaining 10% of subjects in the lower educational stratum would receive a score of 0.95 (0.60 of the first level + 0.30 of the second level + 0.10/2). This calculation was performed for each specific category and provides a continuous score varying from 0 to 1, in which higher values correspond to lower SES^2,41^. For statistical analysis the defined variable was categorized in tertiles labelling the three categories as ‘high’ (1^st^ tertile), ‘medium’ (2^nd^ tertile) and ‘low’ (3^rd^ tertile) SES.

### Life-course SES trajectory

In the EPIC Italy and TILDA cohorts, participants were asked to report their own, their father’s and their partner’s occupational position in a brief questionnaire administered the day of blood collection. Father’s occupational position and highest occupational position (both categorized as ‘low’ and ‘high’) were used as proxies for childhood SES and adulthood SES respectively. The two variables were further combined to create an indicator of the life-course SES trajectory. Further details on the definition of the life-course SES trajectory are given in supplementary material and Stringhini *et al*.^12^.

### Statistical analyses

To avoid bias due to the different distribution of NCD risk factors by cohort, all the SES-AA associations were tested independently for each study and the results meta-analyzed. We used fixed-effect meta-analysis (inverse variance weights) to obtain pooled estimates for SES-AA associations. The I^2^ statistic was used to assess the percentage of variance that is attributable to study heterogeneity^42^. No correction for multiple testing was applied since the SES variables were not mutually independent (Table S8), nor were the two AA measures (Figure S1).

#### Age Acceleration

DNA methylation age was computed according to the algorithm described by Horvath^18^, based on a set of 353 age-associated CpG sites, and the one based on 71 blood-specific age-associated CpG sites described by Hannum *et al*.^19^. Briefly, the DNA methylation age is computed as a weighted average of the age-related CpGs, with weights defined using a penalized regression model (Elastic-net regularization)^18^. The few missing values were imputed using the k-nearest neighboring (KNN) imputation algorithm implemented in the R Bioconductor package *impute* ^43^. Age acceleration (AA) was defined as the difference between epigenetic and chronological age. Positive values of AA (that is epigenetic age is higher than the chronological age) indicate accelerated aging and *vice versa*. Since AA could be correlated with chronological age and WBC percentage, we computed the so-called ‘intrinsic’ AA^20^, defined as the residuals from the linear regression of AA with chronological age and WBC percentages. The latter were estimated using the Houseman algorithm^44^. Intrinsic AA is not dependent on age and WBC by definition. The two AA measures are referred to as ‘Horvath AA’ and ‘Hannum AA’ respectively.

#### SES-AA association

The association of SES with AA was investigated by linear regression models using SES as the predictor and AA as the outcome. The ‘basic adjusted’ model (referred to hereafter also as model 1) included age (continuous), sex, recruitment center (EPIC Italy and TILDA only), incident cancer/cardiovascular event (EPIC Italy only), and sample type (MCCS only) as covariates. In all the analyses the higher SES group was used as the reference to assess associations of low SES with epigenetic AA.

#### Basic adjusted model comparison with NCD risk factors adjusted model

To test for possible reduction in the effect size due to the inclusion of mediators in the model we compared the estimated effect size of model 1 with those of the NCD risk factors adjusted models. The NCD risk factors considered were: smoking status (categorical: never, former, current), BMI (continuous), alcohol intake (categorical: no/moderate, habitual drinkers), Mediterranean Diet Score (ordinal categorical score from 0 to 10, EPIC Italy and MCCS only), and physical activity (ordinal categorical: inactive, moderately inactive, moderately active, active); that were first added to model 1 one-by-one, and finally included all together in the ‘fully adjusted’ model. The reduction in effect size due to the inclusion of mediators in the model was computed as $1-{{\rm{\beta }}}_{{\rm{m}}}/{{\rm{\beta }}}_{1}$; where β_1_ is the effect size of model 1 and β_m_ is the effect size of model 1 plus the mediator(s)^30,45^. Negative value for the effect size reduction occurs when β_1_ < β_m_, and should be interpreted as positive confounding^45^. Confidence intervals and statistical significance of the changes in estimated effect were computed using a block jackknife procedure based on 1,000 resampling^46^.

#### Sensitivity and interaction analyses

Sensitivity analyses were performed to confirm significant associations stratifying the analyses by gender and age groups (younger than, and older than 60 years old) and excluding each time current smokers, habitual drinkers, physically inactive individuals, obese individuals (BMI > 30) and those with unhealthy diet (Mediterranean diet score ≤ 4). The difference in the effect of SES on AA by sex and age class (younger than, and older than 60 years old) was tested by adding the interaction term in the regression analysis. Finally, the associations of SES with AA were further verified after exclusion of incident cancer/cardiovascular cases in EPIC Italy, and individuals exposed to at least one NCD risk factor, leading to the analysis of 1,549 NCD risk factor free individuals.

Raw methylation data may be obtained upon request to giovanni.fiorito@iigm.it. All participants gave written informed consent for their samples to be used in genetic and epigenetic studies of health. This study was reviewed and approved by the HuGeF Ethic Committee. This study was conducted following the principles of the Declaration of Helsinki and its subsequent revisions.

## Electronic supplementary material

Supplemental information
