# Supplementary material for: Social adversity and epigenetic aging: a multi-cohort study on socioeconomic differences in peripheral blood DNA methylation
Source: Sci Rep. 2017 Nov 24;7:16266. doi: 10.1038/s41598-017-16391-5 (PMC5701128; doi:10.1038/s41598-017-16391-5)
Supplement: Supplementary file 1 — Supplemental information [file 41598_2017_16391_MOESM1_ESM.doc]

**Social adversity and epigenetic aging: a multi-cohort study on socioeconomic differences in peripheral blood DNA methylation**

Giovanni Fiorito^a,b,+^, Silvia Polidoro^a,+^, Pierre-Antoine Dugué^c,d^, Mika Kivimaki^e^, Erica Ponzi^f^, Giuseppe Matullo^a,b^, Simonetta Guarrera^a,b^, Manuela B. Assumma^a^, Panagiotis Georgiadis^g^, Soterios A. Kyrtopoulos^g^, Vittorio Krogh^h^, Domenico Palli^i^, Salvatore Panico^j^, Carlotta Sacerdote^k^, Rosario Tumino^l^, Marc Chadeau-Hyam^m^, Silvia Stringhini^n^, Gianluca Severi^a,o^, Allison M. Hodge^c,d^, Graham G. Giles^c,d^, Riccardo Marioni^p^, Richard Karlsson Linnér^q^, Aisling M. O’Halloran^r^, Rose A. Kenny^r^, Richard Layte^r^, Laura Baglietto^s^, Oliver Robinson^m^, Cathal McCrory^r,+^, Roger L. Milne^c,d,+^, Paolo Vineis^a,m,+,*^

**Supplementary methods**

*Subject recruitment and demographic/lifestyle variables acquisition*

*EPIC Italy* -Study participants were drawn from the Italian component of the European Prospective Investigation into Cancer and Nutrition (EPIC) cohort, a large general population cohort consisting of ~520,000 individuals, with standardized lifestyle and personal history questionnaires, measured anthropometric data and blood samples collected for DNA extraction. Smoking habits data were collected at study enrolment through the use of a questionnaire, and participants were categorized as ‘never’, ‘former’ and ‘current’ smokers. Information on dietary habits and alcohol intake were obtained via validated semi-quantitative Food Frequency Questionnaires (FFQ) from which estimated consumption of foods and alcohol in g/day was calculated. Alcohol consumption was treated as a categorical variable: ‘no/moderate’ (less than 28 g/day), ‘habitual’ drinkers (more than 28 g/day). Dietary patterns were assessed by calculating the index of Mediterranean diet score.[^3^](#_ENREF_3) Physical activity was assessed using the Cambridge Physical Activity Index which combines self-reported occupational activity with time participating in cycling and sports. Participants were divided into 4 categories: ‘inactive’ (sedentary job and no recreational activity), ‘moderately inactive’, ‘moderately active’, and ‘active’ (sedentary job with >1 hour of recreational activity per day, standing or physical job with some recreational activity, or a heavy manual job). Height and weight were measured at enrolment with a standardized protocol, and body mass index (BMI) was calculated as the ratio between weight in kg and squared height in meters, treated as a continuous variable.

This study sample includes individuals from five nested case-control studies on breast, colon, and lung cancer, lymphomas, and myocardial infarction. Participants were sampled from the 47,749 participants of the EPIC Italy cohort and included 354 incident breast cancer cases, 169 incident colon cancer cases, 192 incident lung cancer cases, 72 incident lymphoma cases, 292 incident myocardial infarction cases and their 1,079 matched controls. Controls were individually matched on age (±5 years), sex, season of blood collection, center, and length of follow-up. Since the disease diagnoses were made years after blood drawing (only 1.5% of participants developed the disease within two years from blood samples collection), all the subjects were treated as healthy at recruitment and the relationship with SES was investigated irrespective of disease onset (however, 'cancer-case' and 'cardiovascular-case' were included as adjustment variables). In addition, we excluded cases whose diagnosis was made less than one year since blood draw (N=68). For all the individuals a buffy coat sample was collected at recruitment. Overall, after DNA methylation data quality controls and sample filtering 1,803 EPIC Italian subjects were used in this analysis.

*The Melbourne Collaborative Cohort Study* (MCCS) is a prospective cohort study of 41,514 volunteers (~58% women), of which about 99% aged between 40 and 69 years at baseline (between 26 and 76 years overall). At baseline attendance, in 1990-1994, participants completed structured questionnaires that were used to collect anthropometric characteristics (i.e. height and weight) and lifestyle factors including smoking and dietary habits, non-occupational physical activity and alcohol intake. The latter variable was categorized in two groups named ‘no/moderate’ and ‘habitual’ drinkers based on the number of drinks per day (more or less than two drinks), consistently with the measure used in EPIC Italy. Height and weight were measured at baseline according to standardized procedures,[^4^](#_ENREF_4) BMI was calculated as the weight squared height ratio and used as continuous variable. Smoking status, physical activity, and the Mediterranean diet score were assessed following the same procedures described for EPIC Italy.

The present study sample included the controls selected from seven nested case-control studies of breast, colorectal, kidney, prostate, and lung cancer, urothelial cell carcinoma, and B-cell lymphoma, for a total of 3,377 individuals. Individuals with blood samples available only from the follow-up were excluded (N=134). For a large proportion of individuals dried blood spots on Guthrie cards were available while peripheral blood mononuclear cell (PBMC) samples were available for others. The majority of the individuals in the MCCS were Anglo-Saxon (from Australia, or the United Kingdom), whereas about 16% were Mediterranean (Greeks and Italians). Overall, after DNA methylation data quality controls and sample filtering, 2,818 subjects were analyzed in this study.

*The Irish Longitudinal Study on Ageing (TILDA)* is a large prospective cohort study examining the social, economic and health circumstances of 8,175 community-dwelling older adults aged 50 years and over resident in the Republic of Ireland. The sample was generated using a 3-stage selection process and the Irish Geodirectory as the sampling frame. The Irish Geodirectory is a comprehensive listing of all addresses in the Republic of Ireland, which is compiled by the national post service and ordnance survey Ireland. Subdivisions of district electoral divisions pre-stratified by socio-economic status, age, and geographical location, served as the primary sampling units. The second stage involved the selection of a random sample of 40 addresses from within each PSU resulting in an initial sample of 25,600 addresses. The third stage involved the recruitment of all members of the household aged 50 years and over. Consequently, the response rate was defined as the proportion of households including an eligible participant from whom an interview was successfully obtained. A response rate of 62% was achieved at the household level. There were three components to the survey. Respondents completed a computer-assisted personal interview and a separate self-completion paper and pencil module which collected information that was considered sensitive. All participants were invited to undergo an independent health assessment at one of two national centers using trained nursing staff. Blood samples were taken during the clinical assessment with the consent of participants. A more detailed exposition of study design, sample selection and protocol is available elsewhere.[^7^](#_ENREF_7) The present study sample included 500 healthy individuals: 125 for each of the four SES classes: stable professional, any downward mobility, any upward mobility, and stable unskilled (see socioeconomic position assessment). Buffy coat or peripheral blood mononuclear cells (PBMC) samples were available for all the individuals. Overall, after DNA methylation data quality controls and sample filtering, 490 subjects were analyzed in this study.

*Life-course socioeconomic position assessment*

*EPIC Italy* - Participants were asked to report their own, their father’s and their partner’s occupational position in a brief questionnaire administered the day of blood collection. Seven categories were provided: farmers, retailers, office workers, professional occupations, non-skilled manual workers, skilled manual workers, not currently working (including homemakers, retirees, unemployed). Father’s occupational position was categorized as ‘low’ (skilled and non-skilled manual workers, farmers, and retailers) and ‘high’ (professional occupations, and office workers), and was used as a proxy for childhood SES. Household’s highest occupational position was obtained by assigning to each participant the highest occupation from their own occupational position and the partner’s occupational position (the partner’s occupation was assigned to non-working participants) and categorized as done for the father’s occupational position. The household’s highest occupational position was chosen as a proxy for SES due to the high proportion of non-employed women in Italy at the time of sample collection (~45%). The life-course SES trajectory from childhood to adulthood was calculated using information on the father’s occupational position (as the proxy for childhood SES) and the household’s highest occupational position (as the proxy for adulthood SES). Four trajectories were possible: high SES in childhood - high SES in adulthood (stable professional, the reference), high SES in childhood - low SES in adulthood (any downward mobility), low SES in childhood - high SES in adulthood (any upward mobility), and low SES in childhood - low SES in adulthood (stable unskilled).

*TILDA -* The household interview collected data on the respondent’s father’s occupation when they were growing up, before the age of 14. If their father had more than one occupation during this time, TILDA collected data on the most important job (i.e., the one with the highest pay). TILDA also collected information on the respondent’s current occupation (or historic occupation - defined as the job title of the highest paying job they ever held - if they had retired). The coding of occupations followed the Irish Central Statistics Office (CSO) social class schema: (i) professional; (ii) managerial & technical; (iii) non-manual; (iv) skilled manual; (v) semi-skilled; (vi) unskilled; and (vii) all others gainfully occupied but unknown. An eighth category was added to represent those who (viii) never worked. These groupings were then aggregated to create a more manageable number of categories for analysis: (1) Professional / Managerial, (2) Non-manual / Skilled Manual (3) Semi-skilled / Unskilled / Never Worked. The cross-classification of childhood and adulthood social class was used to characterize life-course SES trajectory according to the four categories defined in EPIC. Respondents missing on social class in childhood or adulthood were excluded from the analysis. Respondents who indicated that their father’s occupation was farming were also excluded from the analysis because TILDA did not collect information concerning ownership or size of the farm, which is required to make a social class determination. Exclusion of these cases resulted in a final case base of 5,346 respondents, 3,861 of whom had agreed to provide a blood sample.

*Alternative SES variables*

*MCCS -* In the MCCS the index of relative socioeconomic disadvantage (IRSD) was analyzed as an alternative measure of SES. The IRSD is determined by the Australian Bureau of Statistics which ranks residential areas in Australia according to relative socioeconomic advantage and disadvantage.[^8^](#_ENREF_8) It is based on information from the five-yearly census and is derived from attributes such as low income, low education, unemployment, jobs in relatively unskilled occupations, and other variables reflecting disadvantage. Lower values of the IRSD score correspond to higher SES and *vice versa*. For statistical analyses, we categorized the index in quintiles being the 5^th^ quintile (less disadvantages) the reference category.

*TILDA -* During the household survey, respondents were asked to report all income resulting from full or part-time employment, private or public pensions, and income from other social welfare transfers. Respondents who could not provide an exact figure for income were asked to estimate their income using a banded range: (a) <€10,000 (b) €10,000–<€20,000 (c) €20,000–<€40,000; (d) €40,000–<€70,000 and (e) ≥€70,000. These cases were treated by setting them equal to the mid-point of the banded range. For statistical analyses, we categorized the income in tertiles being the 3^rd^ tertile (higher income) the reference category.

***Microarray protocol***

*EPIC Italy -* For the microarray, DNA samples were extracted from buffy coats using the QIAsymphony DNA Midi Kit (Qiagen, Crawley, UK). Bisulphite conversion of 500 ng of each sample was performed using the EZ-96 DNA Methylation-Gold™ Kit according to the manufacturer’s protocol (Zymo Research, Orange, CA). Then, bisulfite-converted DNA was used for hybridization on the Infinium HumanMethylation 450 BeadChip, following the Illumina Infinium HD Methylation protocol. Briefly, a whole genome amplification step was followed by enzymatic end-point fragmentation and hybridization to HumanMethylation 450 BeadChips at 48°C for 17 h, followed by single nucleotide extension. The incorporated nucleotides were labeled with biotin (ddCTP and ddGTP) and 2,4-dinitrophenol (DNP) (ddATP and ddTTP). After the extension step and staining, the BeadChip was washed and scanned using the Illumina HiScan SQ scanner. The intensities of the images were extracted using the GenomeStudio (v.2011.1) Methylation module (1.9.0) software, which normalizes within-sample data using different internal controls that are present on the HumanMethylation 450 BeadChip and internal background probes. The methylation score for each CpG was represented as a β-value according to the fluorescent intensity ratio representing any value between 0 (unmethylated) and 1 (completely methylated).

*MCCS* – For the majority of the study participants, a blood sample was stored in Guthrie cards, whereas for the remaining individuals, peripheral blood mononuclear cells (PBMC) were extracted from 10 ml blood sample stored in a vacutainer containing sodium heparin. DNA extraction from PBMC were performed using Qiagen mini spin columns (Hilden, Germany), while dried blood spot DNA was extracted using a method developed in-house and described elsewhere.[^9^](#_ENREF_9) Bisulfite conversion (EZ DNA Methylation-Gold kit, Zymo Research, Irvine, CA), quality control analyses and the Infinium HumanMethylation 450 BeadChip DNA methylation assays were performed at the Australian Genome Research Facility as per the manufacturers’ instructions. The bisulfite conversion control dashboard on the Illumina arrays was primarily used to check the conversion success. Also, a bioanalyser run was performed on a subset of samples in each batch to further check the success of the conversion.

*TILDA -* For the microarray, DNA samples were extracted from buffy coats using the QIAGEN GENTRA AUTOPURE LS (Qiagen, Crawley, UK). Bisulphite conversion of 500 ng of each sample was performed using the EZ DNA Methylation-Lightning™ Kit according to the manufacturer’s protocol (Zymo Research, Orange, CA). Then, bisulfite-converted DNA was used for hybridization on the Infinium HumanMethylation 850k BeadChip, following the Illumina Infinium HD Methylation protocol. Briefly, a whole genome amplification step was followed by enzymatic end-point fragmentation and hybridization to HumanMethylation EPIC Chip at 48°C for 17 h, followed by single nucleotide extension. The incorporated nucleotides were labeled with biotin (ddCTP and ddGTP) and 2,4-dinitrophenol (DNP) (ddATP and ddTTP). After the extension step and staining, the BeadChip was washed and scanned using the Illumina HiScan SQ scanner. The intensities of the images were extracted using the GenomeStudio (v.2011.1) Methylation module (1.9.0) software, which normalizes within-sample data using different internal controls that are present on the HumanMethylation 850k BeadChip and internal background probes. The methylation score for each CpG was represented as a β-value according to the fluorescent intensity ratio representing any value between 0 (unmethylated) and 1 (completely methylated).

*DNA methylation data pre-processing and quality controls -* For all the three studies, DNAm data were pre-processed and normalized using in-house software written for the R statistical computing environment, including background and color bias correction, quantile normalization, and BMIQ procedure to remove type I/type II probes bias, as described elsewhere.[^10^](#_ENREF_10) DNAm levels were expressed as the ratio of the intensities of methylated cytosines over the total intensities (β values). Samples were excluded (N=75 in EPIC Italy, N=124 in MCCS, N=4 in TILDA) if the bisulfite conversion control fluorescence intensity was less than 10,000 for both type I and type II probes. Methylation measures were set to missing if the detection p-value was greater than 0.01. Additionally, the set of cross-reactive and/or polymorphic (with minor allele frequency greater than 0.01 in Europeans) CpGs (N=39,238) described by Chen et al.[^11^](#_ENREF_11) was excluded due to the low reliability of methylation measure. Samples (N=99 in EPIC Italy, N=255 in MCCS, N=0 in TILDA) were excluded if the total call rate was less than 95%. Finally, samples (N=4 in EPIC Italy, N=46 in MCCS, and N=6 in TILDA) were excluded if the predicted sex (based on chromosome X methylation) did not match that self-reported, or the reported sample type was inconsistent with the predicted tissue based on the Horvath online methylation age calculator.[^12^](#_ENREF_12)

**Supplementary References**

1 Agnoli, C. *et al.* A priori-defined dietary patterns are associated with reduced risk of stroke in a large Italian cohort. *J Nutr* **141**, 1552-1558, doi:10.3945/jn.111.140061 (2011).

2 Riboli, E. *et al.* European Prospective Investigation into Cancer and Nutrition (EPIC): study populations and data collection. *Public Health Nutr* **5**, 1113-1124, doi:10.1079/PHN2002394 (2002).

3 Agnoli, C. *et al.* Italian Mediterranean Index and risk of colorectal cancer in the Italian section of the EPIC cohort. *Int J Cancer* **132**, 1404-1411, doi:10.1002/ijc.27740 (2013).

4 Haydon, A. M., Macinnis, R. J., English, D. R. & Giles, G. G. Effect of physical activity and body size on survival after diagnosis with colorectal cancer. *Gut* **55**, 62-67, doi:10.1136/gut.2005.068189 (2006).

5 Kinnear, N. J. *et al.* Prostate cancer in men aged less than 50 years at diagnosis. *World J Urol* **34**, 1533-1539, doi:10.1007/s00345-016-1824-4 (2016).

6 Bassett, J. K., Hodge, A. M., English, D. R., MacInnis, R. J. & Giles, G. G. Plasma phospholipids fatty acids, dietary fatty acids, and breast cancer risk. *Cancer Causes Control* **27**, 759-773, doi:10.1007/s10552-016-0753-2 (2016).

7 Whelan, B. J. & Savva, G. M. Design and methodology of the Irish Longitudinal Study on Ageing. *J Am Geriatr Soc* **61 Suppl 2**, S265-268, doi:10.1111/jgs.12199 (2013).

8 Walker, R. & Hiller, J. E. The index of relative socio-economic disadvantage: general population views on indicators used to determine area-based disadvantage. *Aust N Z J Public Health* **29**, 442-447 (2005).

9 Joo, J. E. *et al.* The use of DNA from archival dried blood spots with the Infinium HumanMethylation450 array. *BMC biotechnology* **13**, 23, doi:10.1186/1472-6750-13-23 (2013).

10 Campanella, G. *et al.* Epigenetic signatures of internal migration in Italy. *Int J Epidemiol* **44**, 1442-1449, doi:10.1093/ije/dyu198 (2014).

11 Chen, Y. A. *et al.* Discovery of cross-reactive probes and polymorphic CpGs in the Illumina Infinium HumanMethylation450 microarray. *Epigenetics* **8**, 203-209, doi:10.4161/epi.23470 (2013).

12 Horvath, S. DNA methylation age of human tissues and cell types. *Genome Biol* **14**, R115, doi:10.1186/gb-2013-14-10-r115 (2013).

**Supplementary Figure**

**
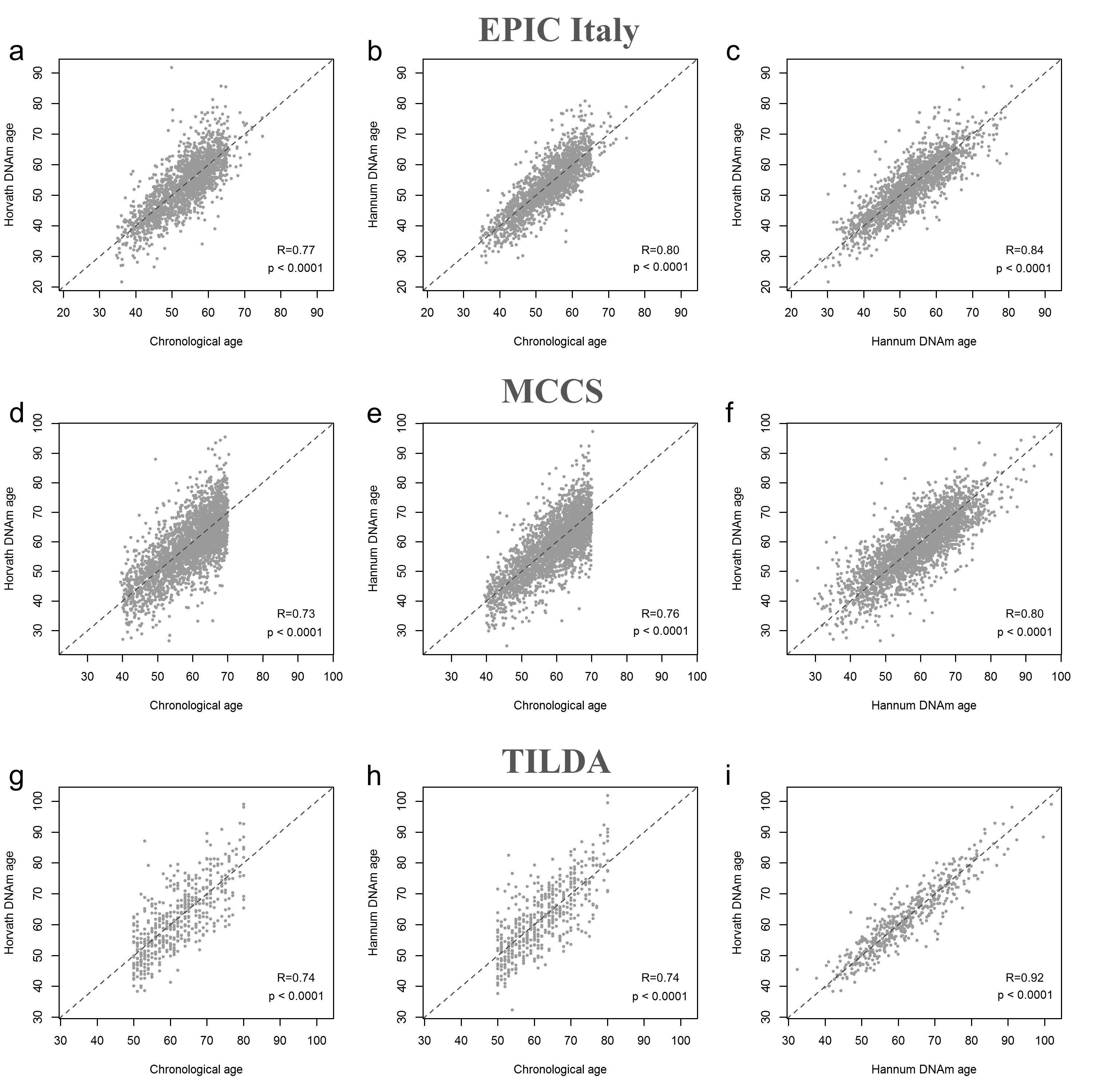
Figure S1. Correlation between chronological and epigenetic age:** Scatterplots indicating the relationship among chronological age, Horvath DNAm age, and Hannum DNAm age in EPIC Italy (Figures S1a, S1b, S1c), MCCS (Figures S1d, S1e, S1f), and TILDA (Figures S1g, S1h, S1i). Dashed lines indicate the bisector of the 1^st^ and 3^rd^ quadrant (in which chronological age is equal to the epigenetic age). Pearson correlation coefficients (R) and p-values for the Pearson correlation test are reported for each comparison.

**Supplementary Tables:**

| **EPIC Italy** | | | | |
| --- | --- | --- | --- | --- |
| **SES** | **N** | **β (95% CI)** | **p** | |
| High | 624 | 0.00 (reference) | **--** | |
| Medium | 643 | 0.58 (-0.31 , 1.48) | 0.20 | |
| Low | 514 | 1.01 (0.13 , 1.90) | 0.02 | |
| Linear trend | 1781 | 0.48 (0.09 , 0.87) | 0.02 | |
| **MCCS** | | | | |
| **SES** | **N** | **β (95% CI)** | **P** | |
| High | 952 | 0.00 (reference) | **--** | |
| Medium | 948 | 0.64 (-0.61 , 1.88) | 0.32 | |
| Low | 917 | 0.43 (-0.96 , 1.81) | 0.55 | |
| Linear trend | 2817 | 0.32 (-0.30 , 0.94) | 0.31 | |
| **TILDA** | | | | |
| **SES** | **N** | **β (95% CI)** | **P** | |
| High | 168 | 0.00 (reference) | **--** | |
| Medium | 158 | 0.65 (-1.06 , 2.36) | 0.46 | |
| Low | 163 | 0.55 (-1.23 , 2.33) | 0.54 | |
| Linear trend | 489 | 0.27 (-0.61 , 1.16) | 0.55 | |
| **Meta-analysis** | | | | |
| **SES** | **N** | **β (95% CI)** | **p** | **I^2*^** |
| High | 1744 | 0.00 (reference) | **--** | **--** |
| Medium | 1749 | 0.61 (-0.06 , 1.28) | 0.07 | 0 |
| Low | 1591 | 0.80 (0.11 , 1.49) | 0.02 | 0 |
| Linear trend | 5087 | 0.41 (0.11 , 0.72) | 0.01 | 0 |
| * I^2^ statistic indicates the percentage of variance that is attributable to study heterogeneity. | | | | |

**Table S1. SES - AA association:** By study area and showing overall meta-analysis of the three study results. Linear regression models with age acceleration (Horvath intrinsic AA) as the outcome and SES as the predictor. Regression models included age, gender, center of recruitment (EPIC Italy and TILDA), case-control status (EPIC Italy only), and sample type (MCCS only).

| **EPIC Italy** | | | | | | | |
| --- | --- | --- | --- | --- | --- | --- | --- |
|  |  | **Model 1 (basic adjustments)** | | | **Full model (adjusted for NCD risk factors)** | | |
| **Life-course SES** | **N** | **β (95% CI)** | **p** | | **β (95% CI)** | **p** | |
| Stable professional | 48 | 0.00 (reference) | -- | | 0.00 (reference) | -- | |
| Downward mobility | 317 | 0.63 (-1.08 , 2.34) | 0.64 | | 0.68 (-1.02 , 2.37) | 0.58 | |
| Upward mobility | 653 | 0.66 (-1.00 , 2.31) | 0.55 | | 0.77 (-0.87 , 2.41) | 0.46 | |
| Stable unskilled | 660 | 1.05 (-0.61 , 2.70) | 0.42 | | 0.98 (-0.66 , 2.62) | 0.43 | |
| Linear trend | 1678 | 0.26 (-0.07 , 0.59) | 0.32 | | 0.21 (-0.13 , 0.54) | 0.46 | |
| **TILDA** | | | | | | | |
|  |  | **Model 1 (basic adjustments)** | | | **Full model (adjusted for NCD risk factors)** | | |
| **Life-course SES** | **N** | **β (95% CI)** | **p** | | **β (95% CI)** | **p** | |
| Stable professional | 123 | 0.00 (reference) | -- | | 0.00 (reference) | -- | |
| Downward mobility | 121 | -0.94 (-2.81 , 0.93) | 0.80 | | -1.51 (-3.50 , 0.47) | 0.49 | |
| Upward mobility | 125 | -0.13 (-1.99 , 1.73) | 0.68 | | -0.71 (-2.69 , 1.27) | 0.84 | |
| Stable unskilled | 121 | 0.91 (-0.96 , 2.79) | 0.22 | | 0.95 (-1.11 , 3.01) | 0.24 | |
| Linear trend | 490 | 0.36 (-0.23 , 0.95) | 0.16 | | 0.33 (-0.32 , 0.98) | 0.23 | |
| **Meta-analysis** | | | | | | | |
|  |  | **Model 1 (basic adjustments)** | | | **Full model (adjusted for NCD risk factors)** | | |
| **Life-course SES** | **N** | **β (95% CI)** | **p** | **I^2^** | **β (95% CI)** | **p** | **I^2^** |
| Stable professional | 171 | 0.00 (reference) | -- | -- | 0.00 (reference) | -- | -- |
| Downward mobility | 438 | -0.09 (-1.35 , 1.18) | 0.90 | 0.30 | -0.24 (-1.54 , 1.05) | 0.71 | 0.60 |
| Upward mobility | 778 | 0.31 (-0.93 , 1.55) | 0.62 | 0 | 0.17 (-1.10 , 1.43) | 0.79 | 0.20 |
| Stable unskilled | 781 | 0.99 (-0.25 , 2.23) | 0.12 | 0 | 0.97 (-0.31 , 2.25) | 0.14 | 0 |
| Linear trend | 2168 | 0.28 (0.00 , 0.57) | 0.05 | 0 | 0.24 (-0.07 , 0.54) | 0.13 | 0 |

**Table S2. Life-course SES - AA association:** Meta-analysis of EPIC Italy and TILDA results. Linear regression models with age acceleration (Horvath intrinsic AA) as the outcome and life-course SES as the predictor. Model 1 included age, gender, center of recruitment, and case-control status (EPIC Italy only); model 2 was as model 1 plus smoking status, BMI, alcohol intake, Mediterranean diet score (EPIC Italy only) and physical activity.

|  | **Hannum AA** | | | | | |
| --- | --- | --- | --- | --- | --- | --- |
|  | **Model 1 (basic adjustments)** | | | **Full model (adjusted for NCD risk factors)** | | |
|  | **β (95% CI)** | **p** | **I^2^** | **β (95% CI)** | **p** | **I^2^** |
| **SES** |  |  |  |  |  |  |
| High | 0.00 (reference) | -- | -- | 0.00 (reference) | -- | -- |
| Medium | 0.71 (-0.14 , 1.55) | 0.10 | 0 | 0.72 (-0.14 , 1.58) | 0.10 | 0 |
| Low | 1.04 (0.16 , 1.93) | 0.02 | 0 | 0.94 (0.04 , 1.84) | 0.04 | 0 |
| Linear trend | 0.44 (0.04 , 0.84) | 0.03 | 0 | 0.38 (-0.03 , 0.78) | 0.07 | 0 |
| **Life-course SES** |  |  |  |  |  |  |
| Stable professional | 0.00 (reference) | -- | -- | 0.00 (reference) | -- | -- |
| Downward mobility | 0.58 (-1.26 , 2.42) | 0.54 | 0 | 0.47 (-1.43 , 2.38) | 0.63 | 0 |
| Upward mobility | 0.44 (-1.36 , 2.23) | 0.63 | 0 | 0.26 (-1.61 , 2.13) | 0.79 | 0 |
| Stable unskilled | 1.85 (0.06 , 3.65) | 0.04 | 0 | 1.77 (-0.13 , 3.67) | 0.07 | 0.13 |
| Linear trend | 0.33 (-0.07 , 0.74) | 0.11 | 0.36 | 0.21 (-0.21 , 0.63) | 0.33 | 0.52 |
|  | **Horvath AA** | | | | | |
|  | **Model 1 (basic adjustments)** | | | **Full model (adjusted for NCD risk factors)** | | |
|  | **β (95% CI)** | **p** | **I^2^** | **β (95% CI)** | **p** | **I^2^** |
| **SES** |  |  |  |  |  |  |
| High | 0.00 (reference) | -- | -- | 0.00 (reference) | -- | -- |
| Medium | 0.06 (-0.87 , 0.99) | 0.90 | 0 | 0.00 (-0.94 , 0.95) | 0.99 | 0 |
| Low | 0.33 (-0.64 , 1.31) | 0.50 | 0.39 | 0.15 (-0.84 , 1.14) | 0.77 | 0.38 |
| Linear trend | 0.13 (-0.33 , 0.60) | 0.57 | 0.64 | 0.05 (-0.42 , 0.51) | 0.85 | 0.60 |
| **Life-course SES** |  |  |  |  |  |  |
| Stable professional | 0.00 (reference) | -- | -- | 0.00 (reference) | -- | -- |
| Downward mobility | 0.30 (-1.64 , 2.24) | 0.76 | 0.40 | 0.23 (-1.80 , 2.26) | 0.82 | 0.48 |
| Upward mobility | 0.39 (-1.50 , 2.29) | 0.68 | 0.17 | 0.09 (-1.90 , 2.09) | 0.93 | 0.51 |
| Stable unskilled | 2.37 (0.47 , 4.27) | 0.01 | 0 | 2.11 (0.08 , 4.14) | 0.04 | 0 |
| Linear trend | 0.63 (0.19 , 1.07) | 0.01 | 0 | 0.44 (-0.02 , 0.91) | 0.06 | 0 |

**Table S3. Meta-analysis of SES - AA association in men:** Linear regression models with SES and life-course SES trajectory as the predictors, and age acceleration (AA, both Hannum and Horvath) as the outcome, in men. The set of confounders contains all the variables included in model 2 except sex.

|  | **Hannum AA** | | | | | |
| --- | --- | --- | --- | --- | --- | --- |
|  | **Model 1 (basic adjustments)** | | | **Full model (adjusted for NCD risk factors)** | | |
|  | **β (95% CI)** | **p** | **I^2^** | **β (95% CI)** | **p** | **I^2^** |
| **SES** |  |  |  |  |  |  |
| High | 0.00 (reference) | -- | -- | 0.00 (reference) | -- | -- |
| Medium | 0.82 (0.00 , 1.63) | 0.05 | 0 | 0.91 (0.08 , 1.74) | 0.03 | 0 |
| Low | 0.93 (0.10 , 1.77) | 0.03 | 0 | 0.95 (0.10 , 1.81) | 0.03 | 0 |
| Linear trend | 0.41 (0.03 , 0.78) | 0.03 | 0 | 0.38 (0.00 , 0.75) | 0.05 | 0 |
| **Life-course SES** |  |  |  |  |  |  |
| Stable professional | 0.00 (reference) | -- | -- | 0.00 (reference) | -- | -- |
| Downward mobility | -0.10 (-1.53 , 1.32) | 0.89 | 0 | -0.17 (-1.61 , 1.28) | 0.82 | 0 |
| Upward mobility | 0.36 (-1.03 , 1.76) | 0.61 | 0 | 0.34 (-1.06 , 1.74) | 0.63 | 0 |
| Stable unskilled | 0.15 (-1.24 , 1.54) | 0.83 | 0 | 0.24 (-1.18 , 1.66) | 0.74 | 0 |
| Linear trend | 0.10 (-0.21 , 0.42) | 0.52 | 0 | 0.11 (-0.21 , 0.43) | 0.49 | 0 |
|  | **Horvath AA** | | | | | |
|  | **Model 1 (basic adjustments)** | | | **Full model (adjusted for NCD risk factors)** | | |
|  | **β (95% CI)** | **p** | **I^2^** | **β (95% CI)** | **p** | **I^2^** |
| **SES** |  |  |  |  |  |  |
| High | 0.00 (reference) | -- | -- | 0.00 (reference) | -- | -- |
| Medium | 1.22 (0.26 , 2.18) | 0.01 | 0 | 1.26 (0.29 , 2.23) | 0.01 | 0 |
| Low | 1.22 (0.23 , 2.21) | 0.02 | 0 | 1.13 (0.12 , 2.13) | 0.03 | 0 |
| Linear trend | 0.52 (0.08 , 0.97) | 0.02 | 0 | 0.44 (-0.02 , 0.89) | 0.06 | 0 |
| **Life-course SES** |  |  |  |  |  |  |
| Stable professional | 0.00 (reference) | -- | -- | 0.00 (reference) | -- | -- |
| Downward mobility | -0.10 (-1.76 , 1.55) | 0.90 | 0 | -0.28 (-1.96 , 1.40) | 0.74 | 0 |
| Upward mobility | 0.44 (-1.19 , 2.07) | 0.60 | 0 | 0.53 (-1.10 , 2.17) | 0.52 | 0 |
| Stable unskilled | 0.14 (-1.49 , 1.77) | 0.87 | 0 | 0.32 (-1.34 , 1.97) | 0.71 | 0 |
| Linear trend | 0.05 (-0.33 , 0.43) | 0.78 | 0 | 0.07 (-0.31 , 0.46) | 0.71 | 0 |

**Table S4. Meta-analysis of SES - AA association in women:** Linear regression models with SES and life-course SES trajectory as the predictors, and age acceleration (AA, both Hannum and Horvath) as the outcome, in women. The set of confounders contains all the variables included in model 2 except sex.

|  | **Hannum AA** | | **Horvath AA** | |
| --- | --- | --- | --- | --- |
|  | **β (95% CI)** | **p** | **β (95% CI)** | **p** |
| SES : sex | 0.00 (-0.55 , 0.55) | 0.99 | -0.39 (-1.02 , 0.25) | 0.23 |
| SES : age | -0.28 (-0.90 , 0.34) | 0.38 | -0.13 (-0.82 , 0.56) | 0.71 |
| Life-course SES : sex | 0.10 (-0.44 , 0.63) | 0.73 | 0.10 (-1.13 , 1.33) | 0.88 |
| Life-course SES : age | 0.09 (-0.51 , 0.69) | 0.77 | 0.54 (-0.71 , 1.78) | 0.40 |

**Table S5. SES-gender and SES-age interaction analyses:** Results for the SES-gender and SES-age interaction analyses. Estimated βs and 95% confidence intervals indicate the differential effect of SES on AA between genders/age groups, and were estimated by adding an interaction term in the linear regression (full adjusted) models.

| **IRSD** | **Hannum AA** | | | |
| --- | --- | --- | --- | --- |
|  | **Model 1 (basic adjustments)** | | **Full model (adjusted for NCD risk factors)** | |
|  | **β (95% CI)** | **p** | **β (95% CI)** | **p** |
| 5^th^ quintile | 0.00 (reference) | -- | 0.00 (reference) | -- |
| 4^th^ quintile | -0.08 (-0.81 , 0.64) | 0.82 | -0.13 (-0.85 , 0.6) | 0.73 |
| 3^rd^ quintile | 0.40 (-0.37 , 1.16) | 0.31 | 0.25 (-0.51 , 1.01) | 0.52 |
| 2^nd^ quintile | 0.80 (0.10 , 1.50) | 0.03 | 0.61 (-0.09 , 1.31) | 0.09 |
| 1^st^ quintile | 0.91 (0.21 , 1.62) | 0.01 | 0.63 (-0.08 , 1.34) | 0.08 |
| Linear trend | 0.26 (0.10 , 0.42) | 0.001 | 0.19 (0.03 , 0.36) | 0.02 |
| **IRSD** | **Horvath AA** | | | |
|  | **Model 1 (basic adjustments)** | | **Full model (adjusted for NCD risk factors)** | |
|  | **β (95% CI)** | **p** | **β (95% CI)** | **p** |
| 5^th^ quintile | 0.00 (reference) | -- | 0.00 (reference) | -- |
| 4^th^ quintile | 0.73 (-0.06 , 1.52) | 0.07 | 0.70 (-0.09 , 1.49) | 0.08 |
| 3^rd^ quintile | 0.90 (0.07 , 1.73) | 0.03 | 0.76 (-0.07 , 1.59) | 0.07 |
| 2^nd^ quintile | 0.65 (-0.11 , 1.41) | 0.09 | 0.47 (-0.29 , 1.24) | 0.23 |
| 1^st^ quintile | 1.10 (0.34 , 1.86) | 0.01 | 0.87 (0.10 , 1.64) | 0.03 |
| Linear trend | 0.22 (0.05 , 0.40) | 0.01 | 0.16 (-0.02 , 0.34) | 0.07 |

**Table S6. IRSD - AA association in MCCS:** Linear regression models with age acceleration (AA) as the outcome and IRDS (in quintiles) as the predictor (full adjusted model). The 5^th^ quintile (less disadvantage: higher SES class) was uses as the reference group.

| **Income** | **Hannum AA** | | | |
| --- | --- | --- | --- | --- |
|  | **Model 1 (basic adjustments)** | | **Full model (adjusted for NCD risk factors)** | |
|  | **β (95% CI)** | **p** | **β (95% CI)** | **p** |
| 3^rd^ tertile | 0.00 (reference) | -- | 0.00 (reference) | -- |
| 2^nd^ tertile | -0.43 (-2.43 , 1.56) | 0.67 | -0.01 (-2.18 , 2.17) | 0.99 |
| 1^st^ tertile | 0.66 (-1.03 , 2.35) | 0.45 | 1.13 (-0.78 , 3.04) | 0.25 |
| Linear trend | 0.27 (-0.56 , 1.10) | 0.52 | 0.52 (-0.42 , 1.45) | 0.28 |
| **Income** | **Horvath AA** | | | |
|  | **Model 1 (basic adjustments)** | | **Full model (adjusted for NCD risk factors)** | |
|  | **β (95% CI)** | **p** | **β (95% CI)** | **p** |
| 3^rd^ tertile | 0.00 (reference) | -- | 0.00 (reference) | -- |
| 2^nd^ tertile | 0.92 (-1.11 , 2.95) | 0.37 | 0.80 (-1.42 , 3.02) | 0.48 |
| 1^st^ tertile | 0.83 (-0.89 , 2.55) | 0.35 | 1.49 (-0.46 , 3.44) | 0.14 |
| Linear trend | 0.45 (-0.40 , 1.30) | 0.30 | 0.75 (-0.21 , 1.70) | 0.13 |

**Table S7. Income - AA association in TILDA:** Linear regression models with age acceleration (AA) as the outcome and income (in tertiles) as the predictor (full adjusted model). The 3^rd^ tertile (individuals with higher income) was uses as the reference group.

| **Study** | **SES indicator 1** | **SES indicator 2** | **χ^2^** | **p** |
| --- | --- | --- | --- | --- |
| **EPIC Italy** | SES | life-course SES trajectory | 396.27 | < 0.0001 |
| **MCCS** | SES | IRSD | 74.72 | < 0.0001 |
| **TILDA** | SES | life-course SES trajectory | 159.02 | < 0.0001 |
|  | life-course SES trajectory | Income | 39.38 | < 0.0001 |
|  | SES | Income | 39.52 | < 0.0001 |

**Table S8. Test for independence among SES indicators:** Chi-squared test for independence among SES indicators used in this study.
